# Modifiers and Readers of DNA Modifications and Their Impact on Genome Structure, Expression, and Stability in Disease

**DOI:** 10.3389/fgene.2016.00115

**Published:** 2016-06-21

**Authors:** Anne K. Ludwig, Peng Zhang, M. C. Cardoso

**Affiliations:** Cell Biology and Epigenetics, Department of Biology, Technische Universität Darmstadt, DarmstadtGermany

**Keywords:** cytosine modifications, Dnmt, epigenetics, hydroxymethylcytosine, MBD, methylcytosine, mouse models, Tet

## Abstract

Cytosine base modifications in mammals underwent a recent expansion with the addition of several naturally occurring further modifications of methylcytosine in the last years. This expansion was accompanied by the identification of the respective enzymes and proteins reading and translating the different modifications into chromatin higher order organization as well as genome activity and stability, leading to the hypothesis of a cytosine code. Here, we summarize the current state-of-the-art on DNA modifications, the enzyme families setting the cytosine modifications and the protein families reading and translating the different modifications with emphasis on the mouse protein homologs. Throughout this review, we focus on functional and mechanistic studies performed on mammalian cells, corresponding mouse models and associated human diseases.

## DNA Modifications and Modifiers

### Cytosine Modifiers: Dnmts

In mammals, the modified cytosine was initially described by Hotchkiss (1948) and was further extensively studied since the 1970s (Razin and Cedar, 1977). Recently, evidence for methylation of adenine has been also reported in mammals (Koziol et al., 2016). Here, we will focus on cytosine modifications in mammals.

DNA cytosine methylation is catalyzed by DNA methyltransferases (Dnmts) that transfer a methyl group from *S*-adenosyl methionine to the fifth carbon of a cytosine residue to form 5-methylcytosine (5mC). The majority of 5mC bases are present in CpG dinucleotides, however, non-CpG methylation was also observed especially in mouse embryonic stem cells (mESCs) and brain tissue (Guo et al., 2014). DNA methylation plays a major role in gene expression, cellular differentiation, genomic imprinting, X-inactivation, inactivation of transposable elements, and embryogenesis.

**FIGURE 1:**
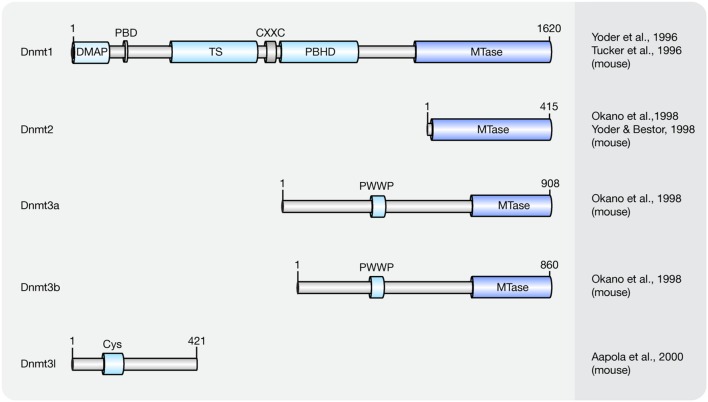
**Schematic representation of the Dnmt protein family.** Shown are domain structures of mouse Dnmt proteins and the initial references. Numbers represent amino acid positions. DMAP, Dnmt1-associated protein binding domain; PBD, proliferating cell nuclear antigen (PCNA)-binding domain; TS, targeting sequence; CXXC, CXXC zinc finger domain; PBHD, polybromo-1 protein homologous domain; MTase, methyltransferase; PWWP, proline-tryptophan-tryptophan-proline motif; Cys, cysteine-rich domain.

Cytosine methylation patterns are mainly established by *de novo* methyltransferases Dnmt3a, Dnmt3b and their regulatory unit Dnmt3l during early embryonic and germ cell development. Once the patterns are established, they are maintained throughout cell generations by Dnmt1 (Bestor et al., 1988; Li et al., 1992). Unlike Dnmt1 and Dnmt3a/3b, Dnmt2 is a RNA methyltransferase rather than a DNA methyltransferase (Okano et al., 1998; Yoder and Bestor, 1998; Goll et al., 2006). A summary of the mouse Dnmt protein family and their domains is shown in **Figure 1** and a summary of the respective knockout mice phenotypes is shown in **Table 1**.

**Table 1:** Phenotypes of initial *Dnmt* knockout mouse models.

Genotype	Phenotype	Reference
*Dnmt1* null	Homozygous knockout *Dnmt1* were stunted, delayed in development, and did not survive past midgestation	Li et al., 1992
*Dnmt3a* null	Knockout mice developed to term and appeared to be normal at birth but most of homozygous mutant mice became runted and died at about 4 weeks of age	Okano et al., 1999
*Dnmt3b* null	No viable *Dnmt3b* knockout mice were recovered at birth	Okano et al., 1999
*Dnmt2* null	Mice homozygous for this *Trdmt1* (formerly *Dnmt2*) knock-out have abnormal RNA methylation while genomic DNA methylation patterns are not detectably altered	Goll et al., 2006
*Dnmt3l* null	Disruption of *Dnmt3l* caused azoospermia in homozygous males and heterozygous progeny of homozygous female died before midgestation	Bourc’his et al., 2001

#### *De novo* DNA Methylation

Overexpression of Dnmt3a and Dnmt3b is capable of methylating both native and synthetic DNA with no preference for hemimethylated DNA (Okano et al., 1999). The domain structure for *de novo* methyltransferases Dnmt3a and Dnmt3b is similar, including a DNA binding domain PWWP domain (Qiu et al., 2002) and a C-terminal catalytic domain (Okano et al., 1999; **Figure 1**). However, several studies showed that the distribution and expression of Dnmt3a and Dnmt3b varies among cell types.

Dnmt3a is expressed relatively ubiquitously and two isoforms of Dnmt3a have been identified. One binds to euchromatic and the other to heterochromatic regions (Okano et al., 1998; Chen et al., 2002). *Dnmt3a* knockout mice developed to term and appeared to be normal at birth but most of the homozygous mutant mice became runted and died at about 4 weeks of age (**Table 1**).

Dnmt3b is highly expressed in embryonic implantation stages, as well as in stem cells and progenitor cells and is the major methyltransferase in early embryogenesis (Watanabe et al., 2002, 2004). Several isoforms were identified and among all isoforms only Dnmt3b1 and Dnmt3b2 possess DNA methyltransferase activity (Aoki et al., 2001). No viable *Dnmt3b* knockout mice were recovered at birth, further highlighting its functions in early embryogenesis (**Table 1**). The major substrates of Dnmt3a/3b are CpGs, but non-CpG methylation activity of Dnmt3a/3b was also detected (Aoki et al., 2001).

Although Dnmt3l does not possess DNA methylation activity (Bourc’his et al., 2001), it strongly interacts with Dnmt3a/3b and enhances their methylation activity (Aapola et al., 2000; Suetake et al., 2004; Hu et al., 2008). However, high expression levels of Dnmt3l are found only in germ cells and early stage embryos but not in somatic cells (Watanabe et al., 2004) indicating that the methylation activity enhancement is cell type and developmental stage dependent. Disruption of *Dnmt3l* caused azoospermia in homozygous males and heterozygous progeny of homozygous female died before midgestation (**Table 1**).

#### Maintenance DNA Methylation

Dnmt1 has a preference for hemi-methylated DNA substrates (Song et al., 2011) and is the enzyme responsible for the maintenance of DNA methylation after DNA replication (Leonhardt et al., 1992). Homozygous knockout *Dnmt1* mice were runted, delayed in development and did not survive past midgestation (**Table 1**). The major isoform of Dnmt1 in mice contains 1620 amino acids and includes an N-terminal regulatory domain and a C-terminal catalytic domain (Tucker et al., 1996; Yoder et al., 1996). However, one isoform lacking the most N-terminal 118 amino acids was shown to accumulate in mouse oocytes (Mertineit et al., 1998).

The Dnmt1-associated protein (DMAP) binding domain is located at the beginning of the N-terminus of Dnmt1 and it recruits DMAP1 to further maintain the heterochromatin state (Rountree et al., 2000). With the contribution of Uhrf1 [ubiquitin-like with plant homeodomain (PHD) and ring finger domains 1], Dnmt1 methylates hemi-methylated DNA generated upon DNA replication by a mechanism encompassing base flipping (Song et al., 2011, 2012).

In most mouse cells, Dnmt1 localizes to the cell nucleus. In fact, Dnmt1 contains several functional nuclear localization sequences within its N-terminal regulatory domain (Cardoso and Leonhardt, 1999). In early embryos (Cardoso and Leonhardt, 1999) and in post-mitotic neurons (Inano et al., 2000) though, it is retained in the cytoplasm. Although highly expressed in mouse embryos, the exclusion of Dnmt1 from nuclei might inhibit DNA methylation conservation after DNA replication (Grohmann et al., 2005), implying that localization of Dnmt1 also regulates its methylation activity. Within the cell nucleus, the distribution of Dnmt1 is cell cycle dependent (Leonhardt et al., 1992). In G1-phase, it is diffusely distributed throughout the nucleoplasm. In early S-phase, its proliferating cell nuclear antigen (PCNA)-binding domain (PBD) targets Dnmt1 to replication sites and in late S-phase, the targeting sequence (TS) further enhances Dnmt1 binding to replicating pericentromeric heterochromatin (Schermelleh et al., 2007; Schneider et al., 2013). In G2-phase, Dnmt1 is *de novo* loaded onto pericentromeric heterochromatin via a replication independent mechanism (Easwaran et al., 2004). Besides its PBD and TS domains, the polybromo-1 protein homologous domain (PBHD) is also involved in targeting Dnmt1 to replication foci (Liu et al., 1998). Between the TS and PBHD domains, a CXXC domain can be found in Dnmt1. The CXXC domain of Dnmt1 occludes access of Dnmt1 catalytic site to non-methylated CpGs and allows Dnmt1 to bind and specifically methylate hemi-methylated CpGs (Song et al., 2011).

### DNA Base Modifications

The stable covalent C–C bond formed between the methyl group and the cytosine is difficult to be directly removed and, therefore, 5mC is thought to be a long-lived epigenetic mark. After DNA replication, Dnmt1 association with the replication machinery ensures the maintenance of the methylation pattern onto the newly synthesized strand. Failure to do so, e.g., by retention in the cytoplasm as mentioned above, leads to gradual passive loss of DNA methylation over cell generations. DNA replication independent (active) loss of global DNA methylation was also observed in some biological processes such as reprogramming of the paternal genome after fertilization (Mayer et al., 2000) and development of primordial germ cells (PGC; Hajkova et al., 2002). The active loss of DNA methylation allows rapid reprogramming of the genome in a short time. Similar observations were made in post-mitotic neurons indicating that active loss of DNA methylation also occurs in somatic cells and might have important roles in the regulation of gene expression (Martinowich et al., 2003).

**FIGURE 2:**
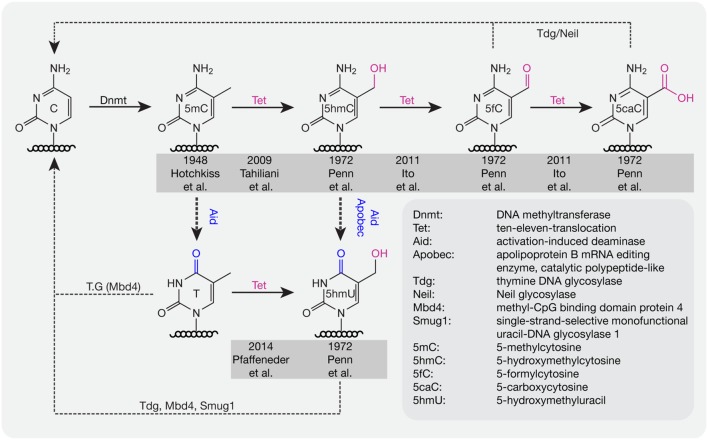
**DNA base modifications with respective enzymes.** Dnmts catalyze the addition of a methyl group to cytosine bases. Tet proteins oxidize methylated cytosines to 5hmC, 5fC, and 5caC in an iterative manner. 5mC and 5hmC can be further deaminated by Aid/Apobec to T and 5hmU. T, 5hmU, 5fC, and 5caC can be removed by the indicated glycosylases. Initial references are indicated.

For several decades, scientists have been interested in identifying pathways or proteins involved in the active loss of DNA methylation. Lacking the evidence to show that C–C bonds can be directly broken in mammals, multi-step processes have been proposed to be involved in the active removal of DNA methylation marks. In 1972, several additional modifications of cytosines were described in rat, mouse, and frog brain tissue including 5-hydroxymethylcytosine (5hmC), 5-formylcytosine (5fC), 5-carboxylcytosine (5caC), and 5-hydroxymethyluracil (5hmU; Penn et al., 1972; **Figure 2**). However, these modifications were considered to be oxidative damage products of DNA (de Rojas-Walker et al., 1995; Tardy-Planechaud et al., 1997). Three decades later, 5hmC was re-discovered in mouse brain tissue (Kriaucionis and Heintz, 2009) and embryonic stem cells (ESCs; Tahiliani et al., 2009). Furthermore, a family of proteins (ten-eleven translocation, TET) was identified that oxidize 5mC to 5hmC both in humans (Tahiliani et al., 2009) and mice (Ito et al., 2010). TET1 was first described in 2003 as a fusion partner of the mixed lineage leukemia (MLL) gene in acute myeloid leukemia (AML; Lorsbach et al., 2003) and 6 years later it was re-discovered as an oxygenase, which can convert 5mC to 5hmC (Tahiliani et al., 2009). Further studies showed that Tet proteins also convert 5hmC to 5fC and 5caC (Ito et al., 2011; Pfaffeneder et al., 2011).

Deaminases such as Aid and Apobec can recognize 5mC and 5hmC and further convert 5mC to thymine (T) and 5hmC to 5hmU. Although the deaminase activity is quite low, it is still a possible pathway for DNA demethylation (Guo et al., 2011). In addition, Tets were also shown to oxidize T to 5hmU in mESCs (Pfaffeneder et al., 2014), which additionally leads to loss of DNA methylation. The oxidation products like 5fC, 5caC, and 5hmU can be recognized and excised by the glycosylases Tdg (Maiti and Drohat, 2011) and Neil (Muller et al., 2014) to create an abasic site on DNA, which is further repaired by enzymes of the base excision repair (BER) pathway. In addition to Tdg, 5hmU can also be recognized by other glycosylases like Mbd4 (Hashimoto et al., 2012b) and Smug1 (Kemmerich et al., 2012). Accordingly, a combination of oxidation, deamination and BER might contribute to the active removal of DNA methylation. In mouse zygotes, the decrease of 5mC and increase of 5hmC suggests that 5hmC might be an intermediate of DNA methylation removal. However, recent studies showed that loss of 5mC mainly happens before S-phase, whereas gain of 5hmC occurred after DNA replication (Amouroux et al., 2016), indicating that besides the conversion of 5mC to 5hmC, other pathways might contribute to methylation removal before DNA replication in mouse zygotes.

### Methylcytosine Modifiers

**FIGURE 3:**
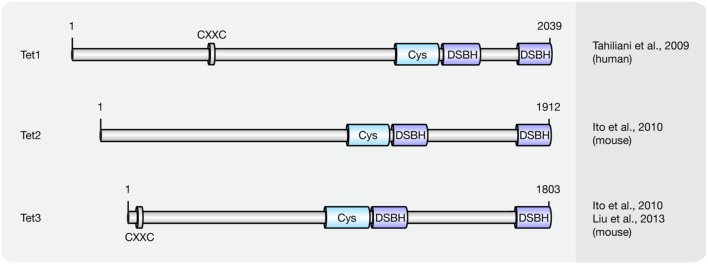
**Schematic representation of the Tet protein family.** Shown are domain structures of mouse Tet proteins and the initial references. Numbers represent amino acid positions. CXXC, CXXC zinc finger domain; Cys, cysteine-rich domain; DSBH, double-stranded β-helix.

Until now three members of the Tet protein family named Tet1 (mouse homolog of human TET1), Tet2 (mouse homolog of human TET2), and Tet3 (mouse homolog of human TET3) have been identified in mice and humans. All three Tets share a conserved C-terminal catalytic domain including a cysteine-rich and a double-stranded β-helix (DSBH) domain, which belong to the cupin-like dioxygenase superfamily; and exhibit iterative iron- and oxoglutarate-dependent oxidation activity (**Figure 3**).

#### Tissue and Genome-Wide Distribution of Tet

During mouse embryo development, Tet3 is highly expressed in oocytes and zygotes. Female mice depleted of *Tet3* in the germ line showed severely reduced fecundity and their heterozygous mutant offspring lacking maternal Tet3 suffer an increased incidence of developmental failure. Since *Tet1*, *Tet2* as well as *Tet1* and *Tet2* double knockout mice are viable, this suggests that *Tet1* and *Tet2* are not essential for mouse development (**Table 2**).

**Table 2:** Phenotypes of initial *Tet* knockout mouse models.

Genotype	Phenotype	Reference
*Tet1* null	Mice are viable, fertile, and grossly normal though some mutant mice have a slightly smaller body size at birth	Dawlaty et al., 2011
*Tet1* null	Animals exhibited abnormal hippocampal long-term depression and impaired memory extinction	Rudenko et al., 2013; Zhang et al., 2013
*Tet2* null	Approximately one-third of *Tet2*^-/-^ and 8% of *Tet2*^+/-^ mice died within 1 year of age because of the development of myeloid malignancies resembling characteristics of chronic myelomonocytic leukemia, myeloproliferative disorder-like leukemia, and myelodysplastic syndrome	Li et al., 2011
*Tet3* null	Female mice depleted of *Tet3* in the germ line show severely reduced fecundity and their heterozygous mutant offspring lacking maternal Tet3 suffer an increased incidence of developmental failure. Oocytes lacking Tet3 also seem to have a reduced ability to reprogram the injected nuclei from somatic cells	Gu et al., 2011
*Tet1* and *Tet2* null	Double deficient mice had reduced 5hmC and increase 5mC levels and abnormal methylation at various imprinted loci. Animals of both sexes were fertile with females having smaller ovaries and reduced fertility	Dawlaty et al., 2014

Tet-mediated 5mC to 5hmC conversion is though involved in reprogramming the paternal genome (Gu et al., 2011; Iqbal et al., 2011; Wossidlo et al., 2011; Zhang et al., 2012) and also in reprogramming donor cell DNA during somatic cell nuclear transfer (Gu et al., 2011). In addition, HIV-1 Vpr binding protein (VprBP)-mediated monoubiquitylation promotes Tet binding to chromatin and enhances 5hmC formation (Nakagawa et al., 2015) in mouse embryos. This process is involved in female germ cell development and genome reprogramming in zygotes (Yu et al., 2013).

During PGC reprogramming, Tet1 and Tet2 are highly expressed (Hackett et al., 2013). However, genome-wide DNA methylation removal is unaffected by the absence of Tet1 and Tet2 and, thus, 5hmC, indicating that the first comprehensive 5mC loss does not involve 5hmC formation. Instead Tet1 and Tet2 have a locus specific role in shaping the PGC epigenome during subsequent development (Vincent et al., 2013). Further studies showed that Tet1 has a critical role in the erasure of genomic imprinting (Yamaguchi et al., 2013) and it controls meiosis by regulating meiotic gene expression (Yamaguchi et al., 2012).

In mESCs, both Tet1 and Tet2, as well as their oxidation product 5hmC are highly abundant (Ito et al., 2010). While Tet2 preferentially acts on gene bodies, Tet1 preferentially acts on promoters and transcription start sites (TSS; Huang et al., 2014). Tet1 and Tet2 double knockout ESCs remained pluripotent, but were depleted of 5hmC and caused developmental defects in chimeric embryos (Dawlaty et al., 2014). During somatic reprogramming, Tet2 is required for 5hmC formation at the *Nanog* locus (Doege et al., 2012). Further studies showed that the recruitment of Tet1 by Nanog facilitates the expression of a subset of reprogramming target genes, such as Oct4 (Costa et al., 2013). Accordingly, Tet1 can replace Oct4 during somatic cell reprogramming in conjunction with Sox2, Klf4, and c-Myc (Gao et al., 2013). The data above indicate that Tet-mediated 5hmC formation is not only important for ESCs differentiation but also for somatic reprogramming.

In mouse brain, 5hmC is a constituent of nuclear DNA (Kriaucionis and Heintz, 2009). Tet1 plays an important role in regulating neural progenitor cell (NPC) proliferation in adult mouse brain (Zhang et al., 2013) and is critical for neuronal activity-regulated gene expression and memory extinction (**Table 2**; Rudenko et al., 2013).

#### Regulation of Tet Activity

Similar to Dnmt1, Tet proteins use a base flipping mechanism to oxidize 5mC, which includes binding of DNA by a Watson–Crick polar hydrogen and van der Waals interactions, flipping out 5mC (Hu et al., 2013; Hashimoto et al., 2014) and oxidation of 5mC to 5hmC (Hashimoto et al., 2015; Hu et al., 2015). Although Tet proteins successively oxidize 5mC to 5caC, recent experimental data showed that, in comparison with 5hmC and 5fC, 5mC is the preferential substrate for Tet2 (Hu et al., 2015). This preference was further confirmed by computer simulations (Lu et al., 2016). In cultured cells, the majority of genomic 5hmC nucleotides are stable (Bachman et al., 2014), indicating that 5hmC is not only involved in loss of DNA methylation, but represents an additional stable epigenetic mark. The global content of 5hmC varies in mouse tissues, does not correlate with 5mC content and rapidly decreases as the cells adapt to cell culture conditions (Nestor et al., 2012). The cell-, tissue-, and developmental stage-specific distribution of 5hmC indicates that the conversion of 5mC to 5hmC is highly regulated.

Although the N-terminal domain (NTD) of Tet proteins was shown to be dispensable for their catalytic activity, it was shown to possess regulatory functions. A CXXC domain, which usually binds specifically to unmethylated CpGs can be found in the N-terminus of Tet1 and Tet3 (Liu et al., 2013). While the CXXC domain of Tet1 cannot bind to DNA *in vitro* (Frauer et al., 2011b), it binds to unmodified C, 5mC- or 5hmC-modified CpGs *in vivo* (Zhang et al., 2010; Xu Y. et al., 2011). Moreover, binding of the CXXC domain to DNA was shown to control DNA methylation levels by preventing unwanted DNA methyltransferase activity in ESCs (Xu Y. et al., 2011) or aberrant methylation spreading into CpG islands (CGIs) in differentiated cells (Jin et al., 2014). The CXXC domain of *Xenopus* Tet3 recognizes non-methylated cytosines in either CpG or non-CpG context, and it is critical for specific Tet3 targeting (Xu et al., 2012). Although Tet2 proteins do not have a CXXC domain, recent studies showed that the ancestral CXXC domain of Tet2 is encoded by a distinct gene named *Idax*. Unlike the CXXC domain of Tet1 and Tet3, the CXXC domain of Idax binds unmethylated CpGs. Through direct protein–protein interactions of Tet2 and Idax, Tet2 is recruited to DNA. Furthermore, Tet2 is degraded by caspase activation, which is triggered by the CXXC of Idax (Ko et al., 2013).

Two parts of the DSBH domain are connected by a potential regulatory spacer region. Although the spacer region was shown to be dispensable for 5mC catalytic activity (Hu et al., 2013), post-translational modifications (PTMs), such as phosphorylation and O-GlcNAcylation were observed in the spacer region (Bauer et al., 2015) indicating that it might exhibit regulatory functions. O-GlcNAc transferase (Ogt) directly interacts with Tet proteins and consequently Tet proteins are GlcNAcylated. The GlcNAcylation does not affect the hydroxylation activity of Tet2 and Tet3, rather Tet2 and Tet3 were shown to promote Ogt activity (Deplus et al., 2013) by enhancing the localization of Ogt to chromatin (Chen et al., 2013; Ito et al., 2014). However, it was shown that Ogt drives Tet3 out of the nucleus further affecting its activity on DNA (Zhang et al., 2014). In mESCs, Ogt is recruited to unmethylated CpG promoters in a Tet1-dependent manner (Vella et al., 2013). In addition to PTMs, mutations within the spacer region of Tet2 were observed in myelodysplastic syndrome (MDS), thus further highlighting the importance of this region (Ko et al., 2010).

*In vivo*, besides PTMs, Tet activity is regulated by protein–protein interactions, such as with Sin3a. In mESCs, the interaction between Sin3a and Tet1 allows Sin3a to repress a subset of Tet1 target genes (Williams et al., 2011). In mouse zygotes, Tet3-mediated 5mC to 5hmC conversion is involved in reprogramming of the paternal but not the maternal genome although they share the same cytoplasm (Mayer et al., 2000). The resistance of the maternal genome to reprogramming is achieved by a protein named developmental pluripotency associated 3 (Dppa3, or PGC7). Dppa3 binds to histone H3K9me2 (Nakamura et al., 2012) and interacts with Tet3 further blocking the activity of Tet3 (Bian and Yu, 2014). Dazl, an RNA-binding protein known to play a key role in germ cell development, was shown to enhance Tet1-mediated 5mC to 5hmC conversion by enhancing Tet1 protein translation (Welling et al., 2015). In addition, growth arrest and DNA damage inducible protein 45 (Gadd45) interacts with Tet1 and Tdg and promotes loss of DNA methylation by enhancing 5fC/5caC removal (Kienhöfer et al., 2015; Li et al., 2015).

Finally, Tet-mediated 5mC to 5hmC conversion was shown to be regulated by Tet cofactors. 2-Ketoglutarate (2-KG), one of the cofactors for Tet oxidation is produced by isocitrate dehydrogenase 1/2 (Idh1/2) *in vivo*. However, mutated Idh1/2 produce 2-hydroxyglutarate, a competitive inhibitor of 2-KG, which can further inhibit 5mC to 5hmC conversion (Konstandin et al., 2011). Vitamin C is a potential cofactor for Tet-mediated oxidation and was shown to enhance Tet activity, which leads to increased global 5hmC in ESCs (Blaschke et al., 2013). ATP was also shown to be involved in regulating Tet activity. *In vitro*, the reaction of Tet-mediated 5mC to 5caC can be enhanced by addition of ATP (He et al., 2011).

#### Hydroxymethylcytosine maintenance

Dnmt1 recognizes hemi-mC DNA and methylates the nascent DNA strand after replication during the S-phase of the cell cycle. However, *in vitro* studies showed a 60-fold decreased binding ability of Dnmt1 to hemi-hmC DNA compared to hemi-mC DNA (Hashimoto et al., 2012a), indicating that hemi-hmC DNA might not be a substrate for Dnmt1. Previous studies showed that Np95 can recognize 5hmC and bind to hemi-hmC DNA (Frauer et al., 2011a), indicating that Np95 might target Dnmt1 to hemi-hmC containing replication forks to maintain hmC after DNA replication. In addition, Dnmt3a and Dnmt3b recognize hemi-hmC DNA (Hashimoto et al., 2012a) and are necessary for methylation maintenance at repeat genomic elements (Chen et al., 2003) suggesting Dnmt3a/3b might play a role in maintaining 5hmC after DNA replication in repeat elements.

*In vivo*, the majority of 5hmC is present in CpG dinucleotides. However, 5hmC has also been observed in non-CpG context, especially in gene bodies (Pastor et al., 2011; Xu Y. et al., 2011). One important role of CpG methylation in gene promoter regions is the repression of gene expression by directly or indirectly preventing interactions between promoter and transcription factors. Hydroxymethylated CpGs might affect binding of transcription factors and/or 5mC readers to DNA.

## DNA Modification Readers

**FIGURE 4:**
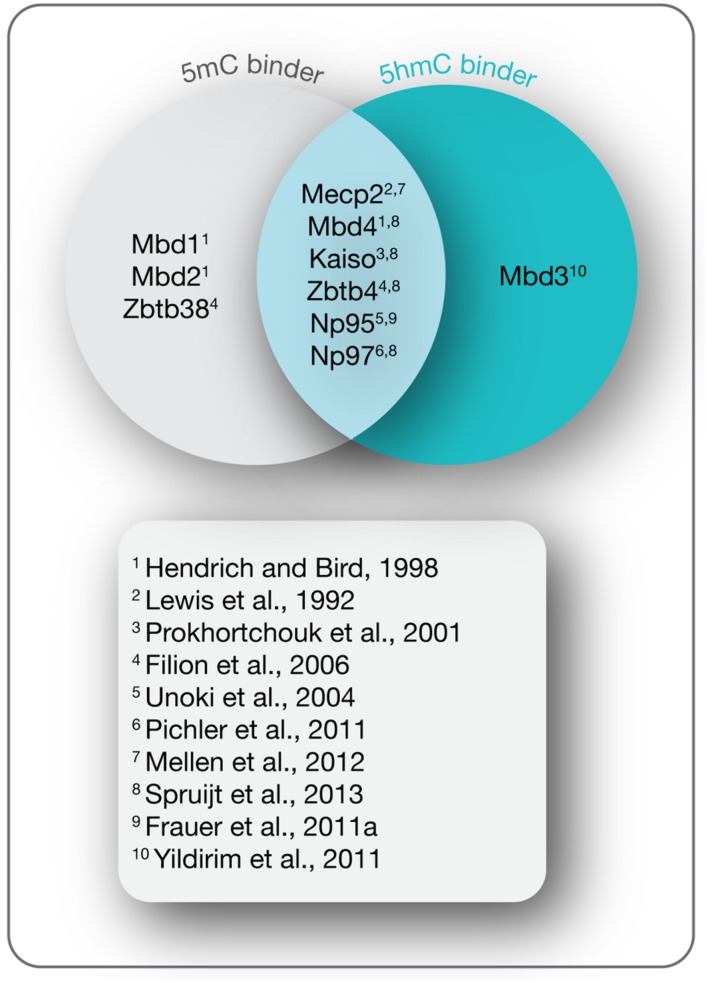
**5-(Hydroxy)methylcytosine readers.** Shown are MBPs that read 5mC and 5hmC as indicated and the initial references.

In mammals, the methylome is specifically read by a variety of proteins known as methyl-CpG binding proteins (MBPs), which based on structural features are further classified into three main families: the methyl-CpG binding domain (MBD) protein family (Lewis et al., 1992; Cross et al., 1997; Hendrich and Bird, 1998; Hendrich and Tweedie, 2003; Laget et al., 2010; Baymaz et al., 2014), the Kaiso protein family (Daniel and Reynolds, 1999; Filion et al., 2006), and the SET and RING (really interesting new gene) finger associated (SRA) domain protein family (Hopfner et al., 2000; Mori et al., 2002). While initially identified as 5mC binding proteins, recent studies indicate that a distinct and dynamic set of MBPs binds the Tet oxidation product 5hmC during differentiation (**Figure 4**; Frauer et al., 2011a; Mellen et al., 2012; Spruijt et al., 2013). Through further interactions with multiple protein partners, MBPs provide a link between cytosine derivatives and functional chromatin states in a temporally and spatially regulated fashion.

### MBD Protein Family

**FIGURE 5:**
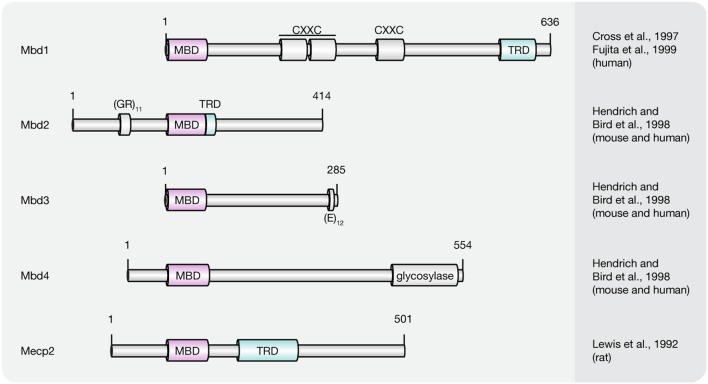
**Schematic representation of the MBD protein family.** Shown are domain structures of mouse MBD proteins and the initial references. Numbers represent amino acid positions. MBD, methyl-CpG binding domain; CXXC, CXXC zinc finger domain; TRD, transcriptional repression domain; GR, glycine/arginine; E, glutamic acid.

Presently, the MBD protein family consists of eleven members (Mecp2, Mbd1–6, SETDB1, SETDB2, TIP5/BAZ2A, and BAZ2B; Lewis et al., 1992; Cross et al., 1997; Hendrich and Bird, 1998; Hendrich and Tweedie, 2003; Laget et al., 2010; Baymaz et al., 2014). All of them share a common protein motif, the 70–85 amino acids long MBD, which enables some, but not all family members, to selectively bind to single methylated CpG dinucleotides. With the exception of Mbd2 and Mbd3, MBD proteins bear little resemblance outside their MBD (Hendrich and Bird, 1998). Instead, MBD proteins comprise several distinct domains that confer unique DNA binding, as well as other functional features. Since this review covers DNA (hydroxy)methylation-dependent processes, we will thereafter focus on MBD family members (**Figure 5**) capable of binding to (hydroxy)methylated CpG dinucleotides, i.e., methyl-CpG binding protein 2 (Mecp2) and methyl-CpG binding domain proteins 1–4 (Mbd1–4).

#### Mecp2

The first protein described to selectively recognize and bind single, symmetrically methylated CpG dinucleotides was Mecp2 (Lewis et al., 1992). It is abundantly expressed in the central nervous system with the highest protein levels in post-mitotic neurons (Akbarian et al., 2001; Traynor et al., 2002; Jung et al., 2003). Of the two alternatively spliced isoforms (Mecp2 e1 and e2), which differ in their N-terminus, Mecp2 e2 was first identified and is, therefore, best characterized (Kriaucionis and Bird, 2004; Mnatzakanian et al., 2004). Although both isoforms distribute differently in developing and post-natal mouse brains, no functional differences have been identified so far (Dragich et al., 2007).

Both Mecp2 variants include two functionally characterized domains, the MBD and the transcriptional repression domain (TRD). While the MBD proved sufficient to direct specific binding to methylated cytosines (Nan et al., 1993), the TRD was originally identified as the region required for transcriptional repression *in vitro* and *in vivo* (Lewis et al., 1992; Nan et al., 1997; Jones et al., 1998; Kaludov and Wolffe, 2000). Circular dichroism and protease digestion analysis revealed that outside these functional domains the full-length protein is largely devoid of secondary structure (Adams et al., 2007). With almost 60% unstructured regions, Mecp2 is reckoned among the intrinsically disordered proteins, which often undergo a disorder-to-order transition upon binding to other macromolecules (Adams et al., 2007). Indeed, recent studies demonstrate that Mecp2 gains secondary structure and acquires substantial thermal stabilization upon binding to DNA (Ghosh et al., 2010). Unlike its name implies DNA binding is, however, not solely mediated via its 5mC specific MBD. Instead, as indicated by the release of Mecp2 upon salt extraction, regions outside the MBD contribute to the overall binding energy through electrostatic interactions (Meehan et al., 1992). As shown by electrophoretic mobility shift assays (EMSAs), these sequence-unspecific DNA binding motifs include the TRD and, based on their relative location to the MBD and TRD, the so-called intervening domain, as well as the C-terminal domain alpha (Ghosh et al., 2010). The NTD of Mecp2 in contrast, contributes indirectly to the overall binding affinity by enhancing the methylation specificity of the MBD through conformational coupling (Ghosh et al., 2010). An analog synergistic increase in DNA binding efficiency was observed through interdomain interactions between the TRD and the C-terminal part of the protein (Ghosh et al., 2010). Similar to the NTD, the C-terminal domain beta (CTD beta) does not directly interact with DNA (Ghosh et al., 2010). Nevertheless, the overall chromatin binding efficiency was lost upon its deletion (Nikitina et al., 2007b). Consistent with this, the CTD beta induced moderate and reproducible shifts with nucleosomal arrays, but not with naked DNA (Ghosh et al., 2010), suggesting that the most C-terminal 192 residues of Mecp2 harbor a chromatin interaction surface (Nikitina et al., 2007b). Indeed, Mecp2 has been shown to interact with histone H3 and, similar to the linker histone H1, binds to nucleosomes close to the linker DNA entry–exit site (Nikitina et al., 2007b). As a result, the entering and exiting linker DNA segments are brought in close proximity to form a stem-like motif (Nikitina et al., 2007a), which bears strong resemblance to structures induced by H1 (Hamiche et al., 1996; Bednar et al., 1998). The modes of chromatin compaction, however, differ significantly from each other. While histone H1 arranges nucleosomes and linker DNA into regular zigzag-shaped chromatin fibers (Woodcock, 2006), Mecp2 forms highly compacted globular structures *in vitro* due to its multiple DNA and chromatin binding domains (Georgel et al., 2003). Accordingly, Mecp2 was shown to induce clustering of pericentric heterochromatin in a dose-dependent manner *in vivo* to establish a locally repressive chromatin environment (Brero et al., 2005; Agarwal et al., 2011). More recently, Szulwach et al. (2011) provided evidence that binding of Mecp2 to methylated CpG dinucleotides may protect 5mC against Tet-mediated oxidation thereby preventing reactivation of silenced genes. The underlying mechanism, however, has so far not been described.

An additional level of regulation is achieved through various protein–protein interactions. While direct homo- and hetero-interactions of Mecp2 and Mbd2 were shown to cross-link chromatin fibers (Becker et al., 2013), physical associations of Mecp2 with the transcriptional co-repressor Sin3a and histone deacetylase 2 (HDAC2) via its TRD contribute to the global heterochromatin architecture through histone hypoacetylation (Jones et al., 1998; Nan et al., 1998). Consequently, Mecp2 deficiency was demonstrated to result in global changes in neuronal chromatin architecture, elevated histone acetylation levels, and increased transcriptional noise in a DNA methylation-dependent manner (Skene et al., 2010; Cohen et al., 2011). A number of other repressive protein partners of Mecp2 have been identified including the co-repressors c-Ski (Kokura et al., 2001), CoREST (Lunyak et al., 2002), and NCoR/SMRT (Stancheva et al., 2003), as well as DNA methyltransferase Dnmt1 (Kimura and Shiota, 2003) and H3K9 methyltransferase (Fuks et al., 2003).

Both, binding of Mecp2 to DNA, as well as interactions with protein partners are affected by PTMs. Neuronal activity induced phosphorylation and dephosphorylation of Mecp2 was shown to modulate its association with promoters of specific genes, as well as with interaction partners (reviewed in Li and Chang, 2014). More recently, poly(ADP-ribosyl)ation of Mecp2 in mouse brain tissue was reported, which anticorrelated with its chromatin binding affinity and clustering ability (Becker et al., 2016). Furthermore, ubiquitylation (Gonzales et al., 2012), SUMOylation (Cheng et al., 2014), acetylation (Zocchi and Sassone-Corsi, 2012), and methylation (Jung et al., 2008) were shown to substantially contribute to the functional versatility of Mecp2.

Another unanticipated level of functional complexity was demonstrated by recent work of Spruijt et al. (2013) who identified Mecp2 as reader of 5hmC in mESC by quantitative mass-spectrometry-based proteomics. Moreover, independent studies of Mellen et al. (2012), revealed Mecp2 as the major 5hmC-binding protein in mouse brain, which moreover turned out to bind both, 5hmC- and 5mC-containing substrates with similar affinity.

Finally, chip-chip analysis using antibodies against MECP2 in a human neuronal cell line demonstrated that around two-third of strongly MECP2 bound promoters were transcriptionally active (Yasui et al., 2007). Subsequent analysis of gene expression patterns in Mecp2 knockout and overexpressing mice concurred that Mecp2 functions as an activator as well as a repressor of transcription (Chahrour et al., 2008).

Hence, the traditional view of Mecp2 as a 5mC-dependent transcriptional silencer may be incomplete and its biology appears far more complicated than previously assumed.

Both, male and female mice lacking Mecp2 (**Table 3**) developed an uncoordinated gait and reduced spontaneous movement between 3 and 8 weeks of age and most died between 6 and 12 weeks (Chen et al., 2001; Guy et al., 2001). Furthermore, most animals developed hind limb clasping, irregular breathing, misaligned jaws and uneven wearing of teeth. Mutant brains were reduced in weight, however, no structural abnormalities or signs of neurodegeneration were detected, suggesting that stability of brain function, not brain development *per se*, is impaired in the absence of Mecp2. Consistent with this hypothesis, re-expression of the Mecp2 gene in Mecp2^lox-Stop/y^ mice proved sufficient to reverse the neurological symptoms of Rett syndrome (RTT), indicating that Mecp2-deficient neurons develop normally and are not irreversibly damaged (Guy et al., 2007). Further microarray analyses revealed that knockout of Mecp2 implicates only minor changes in gene expression (Tudor et al., 2002). Subsequent studies demonstrating increased expression restricted to non-coding RNA in brain of Mecp2-deficient mice (Muotri et al., 2010; Skene et al., 2010), indicated that Mecp2 may not act as a gene-specific transcriptional repressor, but might instead dampen transcriptional noise genome-wide in a DNA methylation-dependent manner (Skene et al., 2010). Accordingly, expression of repetitive elements (Muotri et al., 2010; Skene et al., 2010) as well as retrotransposition of LINE1 was increased in brain of Mecp2-deficient mice (Muotri et al., 2010).

**Table 3:** Phenotype of initial MBP-deficient mouse models.

Genotype	Phenotype	Reference
*Mecp2* null	Rett syndrome-like phenotype. Between 3 and 5 weeks: uncoordinated gait, reduced spontaneous movement, hind limb clasping, irregular breathing, misaligned jaws, uneven wearing of teeth, reduced brain weight, and neuronal cell size. Between 6 and 12 weeks: rapid weight loss and death.	Chen et al., 2001; Guy et al., 2001
*Mbd1* null	Viable and fertile. Impaired spatial learning, decreased neurogenesis, reduced long-term potentiation, decreased genomic stability.	Zhao et al., 2003
*Mbd2* null	Viable, fertile. Maternal nurturing defects: reduced litter size and weight of pups.	Hendrich et al., 2001
*Mbd3* null	Early embryonic lethality	Hendrich et al., 2001
*Mbd4* null	Viable and fertile. Increased number of C:G to T:A transitions at CpG sites.	Millar et al., 2002
*Kaiso* null	Viable and fertile. Reduced tumorigenesis	Prokhortchouk et al., 2006
*Np95* null	Early gestational lethality. Developmental arrest shortly after gastrulation.	Sharif et al., 2007
*Np97* null	Phenotype not described.	Li et al., 2013

#### Mbd1

Mbd1, initially termed PCM1, is expressed in somatic cells and represents the largest member of the MBD family (Cross et al., 1997; Hendrich and Bird, 1998). Similar to Mecp2, Mbd1 contains a MBD and a TRD, which have analog functions to that of Mecp2 (Ng et al., 2000). In addition, depending on the isoform, Mbd1 contains two or three CXXC zinc finger motifs (Fujita et al., 1999; Jorgensen et al., 2004). The most C-terminal one, referred to as CXXC3, is homolog to zinc fingers found in Dnmt1, CpG binding protein CGBP, histone H3K4 methyltransferase MLL and histone H3K36 deacetylases of the Jumonji family JHDM1A and JHDM1B (Jorgensen et al., 2004; Lee and Skalnik, 2005; Tsukada et al., 2006). While CXXC3 was shown to bind unmethylated CpG dinucleotides *in vitro* (Birke et al., 2002; Lee and Skalnik, 2002, 2005; Jorgensen et al., 2004), the remaining zinc finger motifs of Mbd1 lack a conserved glutamine residue and the characteristic KFFG motif necessary for binding to DNA (Jorgensen et al., 2004). Accordingly, Mbd1 isoforms containing the first two CXXC domains preferentially bind methylated DNA via their MBD, whereas isoforms comprising a complete set of zinc fingers have the ability to bind both, methylated and unmethylated substrates (Jorgensen et al., 2004; Baubec et al., 2013).

As a transcriptional repressor, Mbd1 was thus shown to inhibit transcription from both, methylated and unmethylated promoters in reporter gene assays (Fujita et al., 1999; Jorgensen et al., 2004). While methylation-dependent silencing is mediated by the MBD and TRD, suppression of non-methylated reporter constructs required the presence of the CXXC3 domain (Jorgensen et al., 2004). Although, a precise association between Mbd1 and HDACs has not been described, transcriptional repression was partially sensitive to trichostatin A (TSA), an HDAC inhibitor (Ng et al., 2000). In most assays, however, Mbd1 behaved as an HDAC-independent repressor (Ng et al., 2000).

Instead, MBD1 has been found associated with histone H3K9 methyltransferases SETDB1 (Sarraf and Stancheva, 2004) and Suv39h1 (Fujita et al., 2003). Association to SETDB1 mediates transcriptional repression throughout the cell cycle (Sarraf and Stancheva, 2004). During S-phase, however, MBD1 was shown to recruit SETDB1 to the large subunit of chromatin assembly factor CAF-1 to form an S-phase specific complex that mediates methylation of H3K9 in a post-replicative manner (Sarraf and Stancheva, 2004). Accordingly, H3K9 methylation is lost in the absence of MBD1 and results in activation of specific genes, such as p53BP2 (Sarraf and Stancheva, 2004).

MBD1-mediated transcriptional repression and hetero chromatin maintenance was shown to be regulated by SUMOylation (Lyst et al., 2006; Uchimura et al., 2006). In human cells, two E3 SUMO-ligases (PIAS1 and PIAS3) were shown to SUMOylate MBD1 (Lyst et al., 2006). While SUMO1-conjugation blocks the MBD1 and SETDB1 interaction, modification with SUMO2/3 recruits SETDB1 thereby stimulating its repressive function (Uchimura et al., 2006).

Although mice lacking Mbd1 (**Table 3**) developed normally and appeared healthy throughout life, they were impaired in spatial learning, had decreased neurogenesis and reduced long-term potentiation in the dentate gyrus of the hippocampus (Zhao et al., 2003). Moreover, Mbd1-deficient neural stem cells differentiated less and had decreased genomic stability (Zhao et al., 2003).

#### Mbd2

Mbd2 and Mbd3 are the only known members of the MBD protein family with significant sequence similarity beyond the MBD (Hendrich and Bird, 1998) and, thus, are believed to have arisen from an ancient duplication during evolution of the vertebrate lineage (Hendrich and Tweedie, 2003). Consistent with this, a homolog Mbd2/3 like protein was identified in invertebrates, including *Drosophila* (Lyko et al., 2000; Marhold et al., 2004). Despite the high degree of sequence similarity, Mbd3 lacks the amino-terminal extension of Mbd2, which contains a repeat consisting of glycine and arginine residues (Hendrich and Bird, 1998). While both, Mbd2 and Mbd3 contain a C-terminal coiled coil (CC) domain that mediates protein–protein interactions, Mbd3 was shown to comprise an additional glutamic acid repeat at its extreme COOH-terminus (Hendrich and Bird, 1998; Gnanapragasam et al., 2011; Becker et al., 2013).

Mbd2 contains two in-frame start codons, which give rise to Mbd2a and the truncated version Mbd2b, which lacks the first 140 amino acids (Hendrich and Bird, 1998). *In vivo*, however, only Mbd2a, but not Mbd2b, has been detected (Ng et al., 1999). Inclusion of an alternative third exon gives rise to an additional isoform of Mbd2, named Mbd2c, which lacks the C-terminal TRD and CC domain due to an early stop codon (Hendrich and Bird, 1998).

Tethering of Mbd2a near a promoter via a GAL4 DNA binding domain was shown to mediate transcriptional repression that is sensitive to TSA (Ng et al., 1999). Similarly, Mbd2b enhanced transcriptional repression of methylated reporter constructs in co-transfection assays (Boeke et al., 2000). Different from other MBD family members, the sequence required for TRD partially overlapped with the MBD (Boeke et al., 2000), indicating a strong interrelation of methylation binding and transcriptional silencing. In line with this, the TRD directly interacts with the transcriptional repressor Sin3A (Boeke et al., 2000). Moreover, Mbd2 co-purified with a large protein complex known as NuRD (nucleosome remodeling and histone deacetylation), which includes chromatin remodeling ATPase Mi-2, as well as HDAC1 and HDAC2 (Ng et al., 1999; Wade et al., 1999; Zhang et al., 1999; Mahajan et al., 2005; Le Guezennec et al., 2006). EMSAs indicated that Mbd2a directs the NuRD complex, which is implicated in transcriptional silencing, to methylated DNA (Zhang et al., 1999). Finally, immunoprecipitation analysis showed that Mbd2 associates with HDAC1 in mammalian cells and is the long sought methyl-CpG binding component of the 400–800 kDa MeCP1 complex (Meehan et al., 1989; Ng et al., 1999).

Mbd2 was shown to bind 5mC in a manner similar to the isolated MBD of Mecp2 (Hendrich and Bird, 1998; Wade et al., 1999). Binding of oxidative 5mC derivatives, however, has not been observed (Hashimoto et al., 2012a; Mellen et al., 2012; Spruijt et al., 2013).

Mbd2b has also been reported to have DNA demethylase activity (Bhattacharya et al., 1999), but this finding has been questioned (Ng et al., 1999; Wade et al., 1999).

Mbd2-deficient mice (**Table 3**) are viable and fertile, but exhibit a maternal nurturing defect resulting in reduced litter size and weight of pups (Hendrich et al., 2001).

#### Mbd3

The smallest member of the MBD family, coding for a protein of approximately 30 kDa is Mbd3 (Hendrich and Bird, 1998). It appears in a rich diversity of splice variants and is expressed in ESCs as well as somatic tissues (Hendrich and Bird, 1998; Roloff et al., 2003).

DNA binding properties of Mbd3 seem to vary with species. While mammalian Mbd3 is unable to interact with methylated DNA, its amphibian counterpart binds methylated CpG dinucleotides *in vitro* and *in vivo* (Hendrich and Bird, 1998; Wade et al., 1999; Saito and Ishikawa, 2002). Sequence comparison of 5mC binding competent MBD domains revealed two highly conserved residues, which are altered in mammalian Mbd3: a largely solvent exposed tyrosine, as well as an amino-terminal lysine or arginine residue (Ohki et al., 1999; Wakefield et al., 1999; Saito and Ishikawa, 2002).

Despite its inability to recognize 5mC, three different Mbd3 isoforms (Mbd3a–c) that vary in their amino termini were detected within the NuRD repression complex in embryonic stem cells (Zhang et al., 1999; Kaji et al., 2006). ESCs lacking Mbd3-NuRD displayed a severe defect in differentiation that lead to persistent self-renewal even in the absence of leukemia inhibitory factor (Kaji et al., 2006). More recently, depletion of Mbd3 in somatic cells was shown to enhance the reprogramming efficiency of the four Yamanaka factors (Oct4, Sox2, Klf4, and Myc; Luo et al., 2013; Rais et al., 2013). Accordingly, Mbd3 was proposed to play a key role in lineage commitment and pluripotency (Yildirim et al., 2011; Reynolds et al., 2012; Whyte et al., 2012). Contradictory studies using neural and epiblast-derived stem cells, however, indicate a role for Mbd3 in facilitating induction of pluripotency and argue that its function may be context specific (dos Santos et al., 2014).

Binding sites of Mbd3 have been mapped genome-wide in mouse and human cells (Yildirim et al., 2011; Baubec et al., 2013; Gunther et al., 2013; Shimbo et al., 2013). While Yildirim et al. (2011) identified Mbd3 bound to TSS of CpG-rich, hydroxymethylation marked promoters, Baubec et al. (2013) found Mbd3 bound to enhancers independent of CpG density and (hydroxy)methylation status. Further data questioning the interaction of Mbd3 with hydroxymethylated DNA was provided by Spruijt et al. (2013), who did not detect Mbd3 among hydroxymethylation-specific readers.

Although both, Mbd2 and Mbd3 associate with the NuRD complex, the two MBD containing complexes appear to have no functional overlap since knockout of Mbd3 in mice is embryonic lethal, whereas Mbd2-deficient mice are viable and fertile (Hendrich et al., 2001; **Table 3**).

#### Mbd4

Mbd4, also referred to as MED1 (Bellacosa et al., 1999), is the only known member of the MBD protein family not associated with HDAC activity (Hendrich and Bird, 1998). Instead, several lines of evidence suggest that Mbd4 plays a role in DNA repair (Bader et al., 1999; Bellacosa et al., 1999; Hendrich et al., 1999; Riccio et al., 1999; Petronzelli et al., 2000; Millar et al., 2002). In addition to its MBD, Mbd4 contains a C-terminal catalytic domain that is highly homologous to bacterial DNA damage specific endonucleases that exhibit glycosylase activity during BER (Michaels et al., 1990; Hendrich and Bird, 1998). Accordingly, Mbd4 was shown to remove thymine or uracil from mismatched CpG sites through glycosidic bond cleavage. As genomic G/T mismatches are the expected product of 5mCpG deamination, Mbd4 has been designated a methylation specific DNA repair enzyme (Hendrich et al., 1999; Petronzelli et al., 2000; Hashimoto et al., 2012b). Furthermore, Mbd4 has been implicated in DNA demethylation as it was shown *in vitro* to excise 5hmU, the deamination product of 5hmC (Hashimoto et al., 2012b).

Knockout and rescue experiments in embryonic stem cells, however, demonstrated that oxidation-dependent reactivation of methylated reporter genes is mediated by the action of thymine DNA glycosylase (TDG), but not by Mbd4 (Muller et al., 2014). Accordingly, deamination of 5hmC to 5hmU and subsequent excision by Mbd4 does not play a major role in ESCs (Hashimoto et al., 2012b). A contribution of Mbd4 to Tet-initiated DNA demethylation in NPCs, however, cannot be excluded, since Mbd4 was shown to bind to 5hmC at this developmental stage (Spruijt et al., 2013).

Mice lacking Mbd4 are viable and fertile (Millar et al., 2002). However, compared to wild-type mice, Mbd4 knockout lead to a 3.3-fold higher number of C:G to T:A transitions at CpG sites (Millar et al., 2002). Moreover, Mbd4-/- mice that were made heterozygous for the Min allele of the adenomatous polyposis coli gene (ApcMin), which pre-disposes mice to develop spontaneous intestinal neoplasia (Su et al., 1992), showed markedly reduced survival compared to Mbd4+/+ controls. Accordingly, Mbd4 plays an important role in the repair of 5mC deamination at mCpGs. The relatively mild phenotype of Mbd4 knockout mice (**Table 3**), however, suggests that its absence might be compensated for by other glycosylases, such as TDG.

### Kaiso Protein Family

**FIGURE 6:**
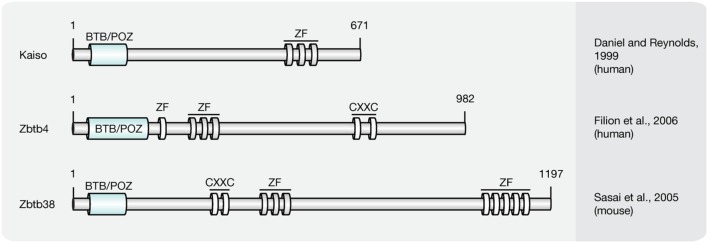
**Schematic representation of the Kaiso-like protein family.** Shown are domain structures of mouse Kaiso-like proteins and the initial references. Numbers represent amino acid positions. BTB/POZ, broad complex, tramtrack and bric a brac/poxvirus and zinc finger domain; ZF, zinc finger; CXXC, CXXC zinc finger domain.

Members of the Kaiso-like protein family (**Figure 6**) present a second class of proteins capable of binding specifically to methylated DNA (Filion et al., 2006). In contrast to members of the MBD protein family, Kaiso, Zbtb4, and Zbtb38 contain a conserved BTB/POZ (Bric-a-brac, tramtrack, broad complex/poxvirus and zinc finger) domain involved in protein–protein interactions and three Kruppel-like C2H2 zinc finger motifs, of which two were found essential for binding to methylated DNA (Filion et al., 2006). Similar to MBD proteins, members of the Kaiso family function as HDAC-dependent transcriptional repressors (Sasai et al., 2005). Several lines of evidence, however, including their variable binding modes, protein partners and expression patterns, suggest that Kaiso-like proteins have different biological functions (Daniel and Reynolds, 1999; Kiefer et al., 2005; Park et al., 2005; Filion et al., 2006).

While Kaiso was shown to require at least two methylated CpG dinucleotides, a single mCpG proved sufficient for efficient binding of the Zbtb4 and Zbtb38 proteins (Prokhortchouk et al., 2001; Filion et al., 2006). Besides its ability to bind methylated DNA, *in vitro* synthesized Kaiso was shown to interact specifically with an unmethylated consensus sequence, the Kaiso binding site (KBS, TCCTGCNA), which can be found at promoters of Wnt target genes (Daniel and Reynolds, 1999; Park et al., 2005). Accordingly, the xWnt11 gene, a target of non-canonical Wnt signaling, was shown to be regulated by Kaiso in *Xenopus* (Daniel and Reynolds, 1999; Prokhortchouk et al., 2001). Moreover, Kaiso-mediated repression of non-canonical and canonical Wnt targets was repressed by interactions with p120-catenin (Kim et al., 2004), as it competes with DNA for the access to the Kaiso zinc finger domains (Daniel et al., 2002). The ability to bind unmethylated KBS sequences is shared by Zbtb4. Zbtb38, however, was shown to interact with the E-box motif (CACCTG) of the rat tyrosine hydroxylase gene promoter (Kiefer et al., 2005), but failed to bind a labeled KBS probe (Filion et al., 2006). More recently, Kaiso was found to bind 5hmC in NPCs and Zbtb4 was pulled down with hydroxymethylated DNA from brain tissue (Spruijt et al., 2013). The 5hmC binding domains, as well as the biological function, however, remain to be determined.

Kaiso-like proteins contain a BTB/POZ domain, which facilitates interaction with different sets of co-repressors and mediate transcriptional repression.

Kaiso was shown to recruit the NCoR complex to promoters of target genes to introduce histone hypoacetylation, as well as H3K9 methylation (Yoon et al., 2003). Moreover, Kaiso was identified as component of an alternative MeCP1 complex in NIH3T3 cells (Prokhortchouk et al., 2001). Zbtb38 was found to interact with the co-repressors CtBPs (C-terminal binding proteins), which include HDAC, methyltransferase, and demethylase activities (Sasai et al., 2005; Zocchi and Sassone-Corsi, 2012). Zbtb4 was shown to associate with the Sin3A/HDAC complex to repress expression of p21^CIP1^ in response to stimuli that activate p53 (Weber et al., 2008).

Kaiso-like proteins exhibit diverging expression patterns. While Kaiso is ubiquitously expressed, *Zenon*, the rat homolog of *ZBTB38*, is primarily transcribed in brain and neuroendocrine tissues (Kiefer et al., 2005). For Zbtb4, in contrast, high expression levels were identified in brain, lung, kidney, muscle, and heart (Filion et al., 2006).

Kaiso-null mice (**Table 3**) are viable and fertile, with no detectable changes in gene expression profiles or developmental abnormalities. However, when crossed with tumor-susceptible Apc(Min/+) mice, Kaiso-deficient animals showed resistance to intestinal cancer (Prokhortchouk et al., 2006).

### SRA Domain Protein Family

**FIGURE 7:**
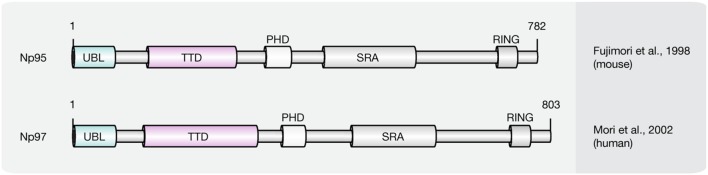
**Schematic representation of the SRA domain protein family.** Shown are domain structures of mouse SRA domain proteins and the initial references. Numbers represent amino acid positions. UBL, ubiquitin-like domain; TTD, tandem tudor domain; PHD, plant homeodomain; SRA, SET, and RING finger associated domain; RING, really interesting new gene.

Recent studies implicate that yet another protein fold, the SRA domain could read DNA (hydroxy)methylation marks *in vitro* and *in vivo* (Unoki et al., 2004; Johnson et al., 2007; Woo et al., 2007; Frauer et al., 2011a; Spruijt et al., 2013). In mammals, two SRA domain-containing proteins (**Figure 7**), Np95 (mouse homolog of human ICBP90, gene name *UHRF1*) and Np97 (mouse homolog of human NIRF, gene name *UHRF2*), have been characterized (Unoki et al., 2004; Woo et al., 2007; Zhang et al., 2011). While Np95 was first discovered during the generation process of antibodies against murine thymic lymphoma (Fujimori et al., 1998), NIRF was identified through screenings for PCNP (PEST containing nuclear protein) interaction partners (Mori et al., 2002).

Besides the eponymous SRA domain, ICBP90 contains at least four additional functional motifs (Hashimoto et al., 2009): an N-terminal ubiquitin-like domain (Ubl, or NIRF_N); a tandem Tudor domain (TTD) that binds histone H3 tails di/tri-methylated at lysine 9 (H3K9me2/3; Karagianni et al., 2008; Papait et al., 2008; Rottach et al., 2010); a PHD, which binds (un)modified histones; and a C-terminal RING, which exhibits ubiquitin E3 ligase activity.

ICBP90 and Np95 play a critical role in epigenetic inheritance and maintenance of DNA methylation (Bostick et al., 2007; Sharif et al., 2007). Accordingly, ICBP90/Np95 was shown to colocalize with PCNA during S phase and to interact with Dnmt3a, Dnmt3b and several histone-modifying enzymes like HDAC1, as well as histone methyltransferase G9a (Achour et al., 2009; Kim et al., 2009; Meilinger et al., 2009). Moreover, besides its ability to bind and flip out hemi-methylated DNA, the SRA domain of ICBP90 was shown to target Dnmt1 to replicating pericentric heterochromatin for maintenance methylation (Bostick et al., 2007; Arita et al., 2008; Avvakumov et al., 2008; Hashimoto et al., 2008; Papait et al., 2008). In addition, ICBP90 was shown to bind histone H3K9me2/3 via its TTD, thus connecting repressive histone marks with DNA methylation (Rottach et al., 2010; Nady et al., 2011; Rothbart et al., 2012). The PHD of ICBP90, on the other hand, was found associated with the N-terminal tail of histone H3 (Papait et al., 2007; Hu et al., 2011; Rajakumara et al., 2011; Wang et al., 2011; Arita et al., 2012; Cheng et al., 2013). More recently, the SRA domain of Np95 was demonstrated to bind 5hmC and 5mC containing DNA substrates with similar affinity *in vitro* (Frauer et al., 2011a). Consistent with this, Np95 was identified as 5hmC reader in mESCs and NPCs. In mouse brain tissue, however, association with 5hmC remained undetected likely due to its low expression levels. Although the structure of NIRF, the second member of the SRA domain protein family, is closely related to ICBP90, both proteins possess significantly different expression patterns. While ICBP90 is mainly expressed in proliferating cells (Fujimori et al., 1998), NIRF protein levels increase during differentiation (Pichler et al., 2011). NIRF binds hemi-methylated DNA and H3K9me2/3 containing heterochromatin marks in a cooperative manner, whereby localization and *in vivo* binding dynamics of NIRF, were shown to require an intact TTD and depend on H3K9me3 but not on DNA methylation (Pichler et al., 2011). While Np95 was shown to bind 5hmC in mESCs and NPCs, the interaction of Np97 and 5hmC was specific for NPCs. Furthermore, Np97 exhibited higher binding affinity for 5hmC than for 5mC in NPCs (Spruijt et al., 2013). Finally, Np97 was proposed to promote repetitive oxidation of 5mC by Tet proteins, since the levels of the oxidative cytosine derivatives 5hmC, 5fC and 5caC were increased upon coexpression of Np97 and Tet1 in HEK293T cells (Spruijt et al., 2013). Consequently, Spruijt et al. (2013), hypothesized that flipping of the modified base, as previously described for Np95, may enhance the accessibility of Tet enzymes to the hydroxymethylated base, whereby further oxidation is promoted.

Furthermore, ectopic Np97 was unable to rescue DNA methylation defects observed in *Np95-/-* ESCs. Neither DNA methylation levels, nor pericentric heterochromatin localization of Dnmt1 in S-phase could be restored upon overexpression of Np97 arguing for functional differences between both proteins (Pichler et al., 2011). NIRF was found to interact with cell cycle proteins including cyclins, cyclin-dependent kinases (CDKs), retinoblastoma protein (pRB), p53, PCNA, HDAC1, DNMTs, and G9a (Mori et al., 2012). It was shown to ubiquitinate cyclins D1 and E1, and to induce G1 arrest. Accordingly, NIRF was proposed to link the cell cycle regulatory network with the epigenetic landscape (Mori et al., 2012).

While knockout of Np95 leads to developmental arrest shortly after gastrulation and early gestational lethality (Sharif et al., 2007), the phenotype of Np97 null mice has not been analyzed (Li et al., 2013; **Table 3**).

## Role of 5mC Writers, Readers, and Modifiers in Disease

Mutations in proteins involved in writing, reading, and modifying the epigenetic landscape have been implicated in various severe human disorders. Due to their high sequence (**Table 4**) and functional similarity (Kumar et al., 1994; Hendrich and Bird, 1998; Mori et al., 2002; Filion et al., 2006; Bostick et al., 2007; Ito et al., 2010; Qin et al., 2011), we, hereafter, summarize the state-of-the-art regarding the role of the human orthologs of the aforementioned mouse Dnmts, Tets, and MBPs in human diseases.

**Table 4:** Comparison of human proteins and their mouse orthologs.

Mouse protein	Human protein	Amino acid similarity (%)
Dnmt1 (1620 aa)	DNMT1 (1632 aa)	76
Dnmt2 (415 aa)	DNMT2 (391 aa)	77
Dnmt3a (908 aa)	DNMT3A (912 aa)	96
Dnmt3b (860 aa)	DNMT3B (853 aa)	80
Dnmt3l (421 aa)	DNMT3L (387 aa)	56
Tet1 (2039 aa)	TET1 (2136 aa)	50
Tet2 (1912 aa)	TET2 (2002 aa)	55
Tet3 (1803 aa)	TET3 (1795 aa)	89
Mecp2 (501 aa)	MECP2 (498 aa)	94
Mbd1 (636 aa)	MBD1 (605 aa)	68
Mbd2 (414 aa)	MBD2 (411 aa)	94
Mbd3 (285 aa)	MBD3 (291 aa)	92
Mbd4 (554 aa)	MBD4 (580 aa)	58
Kaiso (671 aa)	KAISO (672 aa)	84
Zbtb4 (982 aa)	ZBTB4 (1013 aa)	85
Zbtb38 (1197 aa)	ZBTB38 (1195 aa)	81
Np95 (782 aa)	ICBP90 (806 aa)	72
Np97 (803 aa)	NIRF (802 aa)	90

### DNMT Proteins in Disease

Since *Dnmt1* knockout is embryonic lethal in mice, it is unlikely to expect a human disease linked to a DNMT1 catalytic domain mutation. But mutations in the regulatory domain of DNMT1 were found (**Table 5**). Mutations in the TS domain of DNMT1 cause neurodegeneration like hereditary sensory autonomic neuropathy with dementia and hearing loss (HSAN1E; Klein et al., 2011) and autosomal dominant cerebellar ataxia, deafness and narcolepsy (ADCA-DN; Winkelmann et al., 2012). Mutations of Y495C, Y495H, D490E-P491Y (Klein et al., 2011, 2013) in exon 20 cause HSAN1E. Those mutations caused premature degradation of mutant proteins, reduced methyltransferase activity and impaired heterochromatin binding during G2 phase leading to global hypomethylation and site-specific hypermethylation (Klein et al., 2011). ADCA-DN is a polymorphic disorder first described in 1995 in a Swedish pedigree. Unlike mutations in HSAN1E located in exon 20, mutations in ADCA-DN including A570V, G605A, and V606F were found in exon 21 of the *DNMT1* gene.

**Table 5:** Summary of disease-related DNMT and TET mutations.

Protein	Disease	Alteration	Reference
DNMT1	Hereditary sensory autonomic neuropathy with dementia and hearing loss (HSAN1E)	Y495C, Y495H, D490E-P491Y	Klein et al., 2011, 2013
DNMT1	Autosomal dominant cerebellar ataxia, deafness and narcolepsy (ADCA-DN)	A570V, G605A, and V606F	Winkelmann et al., 2012
DNMT3A	Acute myeloid leukemia (AML) myelodysplastic syndrome (MDS)	R882 and frameshift, nonsense and splice site mutations	Ley et al., 2011; Walter et al., 2011
DNMT3A	Overgrowth syndrome	Mutations interfere with domain–domain interactions and histone binding	Tatton-Brown et al., 2014
DNMT3B	Immunodeficiency, centromeric region instability, facial anomalies syndrome (ICF) syndrome	Mutations in catalytic domain	Hansen et al., 1999; Xu et al., 1999
TET1	AML	Ten-eleven translocation that gives rise to a MLL-TET1 fusion	Lorsbach et al., 2003
TET2	AML, MDS, and myeloproliferative neoplasms	Mutations mostly in catalytic domain	Abdel-Wahab et al., 2009

Mutations in DNMT3A were found in *de novo* AML and are associated with poor survival (**Table 5**; Ley et al., 2011). The most frequent mutation occurred in amino acid R882, however, frameshift, nonsense and splice site mutations were also reported (Ley et al., 2011). Mutations of DNMT3A are not only observed in AML patients, but also in MDS. Similar to mutations leading to AML, amino acid R882 located in the methyltransferase domain of DNMT3A is the most common mutation site (Walter et al., 2011). Unlike in AML and MDS, most mutations in overgrowth syndrome do not directly affect the catalytic activity of DNMT3A, but interfere with domain–domain interactions and histone binding, which further affect the activity of DNMT3A (Tatton-Brown et al., 2014).

ICF syndrome (immunodeficiency, chromosomal instability, and facial anomalies), a human genetic disorder is caused by DNMT3B mutations (**Table 5**; Hansen et al., 1999; Xu et al., 1999). Several mutations were identified and most mutations are located in the catalytic domain of DNMT3B and directly affect the activity of DNMT3B (Xu et al., 1999). However, mutations, which do not directly affect its catalytic activity were also observed in ICF syndrome. Two mutations, A766P and R840Q displayed similar methylation activity than the wild-type enzyme but lost the ability to interact with DNMT3L, which further leads to loss of activity *in vivo* (Xie et al., 2006). Direct or indirect loss of DNMT3B activity consequently decreased satellite DNA methylation in ICF syndrome patients, indicating that DNMT3B is involved in maintaining genome stability.

5mC, the product of DNMTs is related to tumorigenesis. It was shown that the genome of cancer cells is globally hypomethylated relative to their normal counterparts. Usually, hypomethylation leads to gene activation. In cancer cells, the activation of genes is caused by hypomethylation of nearby CGIs, which are silenced in somatic tissues by DNA methylation (Strichman-almashanu et al., 2002). Satellite sequences and repetitive sequences such as LINE1, SINE, IAP, and Alu elements are silenced mainly by DNA methylation in normal cells. However, in tumor cells, hypomethylation of L1 promoter was detected and the activation of L1 might promote chromosomal rearrangements and genome instability (Suter et al., 2004). Although the cancer genome is hypomethylated, several studies showed that Dnmts are upregulated in cancer cells (Ahluwalia et al., 2001; Lin et al., 2007; Roll et al., 2008), suggesting that demethylation enzymes might be additionally involved in loss of DNA methylation in cancer.

### TET Proteins in Disease

*MLL* gene is located in 11q23 and is the most frequent cytogenetic finding in AML. In AML, *MLL* is translocated to chromosome 10 as a fusion with the *TET1* gene. The MLL-TET1 fusion protein contains the AT hooks, subnuclear localization domains, and the CXXC domain of MLL and the C-terminus of TET1 (**Table 5**; Lorsbach et al., 2003). The function of MLL-TET1 fusion protein is still unknown, but it was showed that TET1 is involved in MLL-rearranged leukemia. *TET1* is a direct target of the MLL-fusion protein and is significantly upregulated in MLL-rearranged leukemia, leading to a global increase 5hmC, thus playing an oncogenic role (Huang et al., 2013).

In myeloproliferative neoplasms, mutations of TET2 but not TET1 and TET3 were observed (**Table 5**; Abdel-Wahab et al., 2009). Mutations of TET2 were also observed in AML with varied frequency and most of them occurred in the catalytic domain of TET2. In AML, TET2 mutations correlate with genomic 5hmC level (Konstandin et al., 2011). TET2 is one of the most frequently mutated genes in MDS. Mutations of TET2 were detected in most of the bone marrow cells in MDS and these mutations contribute to the malignant transformation of bone marrow cells (Langemeijer et al., 2009), which consequently displayed uniformly low levels of 5hmC in genomic DNA compared to bone marrow samples from healthy controls (Ko et al., 2010).

Besides the hematopoietic malignancies, 5hmC levels are also changed in solid tumors. 5hmC level were profoundly reduced in glioma, colon cancer, breast cancer, and melanoma compared to normal tissues (Haffner et al., 2011; Jin et al., 2011; Li and Liu, 2011; Xu W. et al., 2011; Kraus et al., 2012).

Unlike in cancer, in the hippocampus/parahippocampal gyrus (HPG) of preclinical and later-stage Alzheimer’s disease patients, significantly increased levels of TET1, 5mC, and 5hmC were observed. In contrast, levels of 5fC and 5caC were significantly decreased in the HPG of these patients (Bradley-Whitman and Lovell, 2013). This indicates that DNA methylation might play an important role in memory-related disease.

### MBPs in Disease

As readers and translators of epigenetic information, alterations in MBP sequences affect the precisely coordinated link between DNA methylation, histone modification and higher order chromatin structure.

Mutations in the X-linked *MECP2* gene give rise to RTT (**Table 6**), a late onset (6–18 months post-birth) debilitating neurological disease that affects 1 in 10,000–15,000 female live births (Hagberg et al., 1983; Amir et al., 1999). After a period of normal development (6–18 months), RTT patients usually lose speech and acquired motor skills (Hagberg et al., 1983). They are aﬄicted with seizures, autism, loss of motor coordination, abnormal breathing and develop stereotypical, repetitive hand movements (Hagberg et al., 1983). After the initial regression, however, conditions often stabilize and allow viability until adulthood (Rett, 1966; Hagberg et al., 1983).

**Table 6:** Summary of disease-related MBP alterations.

Protein	Disease	Alteration	Reference
MECP2	Rett syndrome	Causal MECP2 mutations of Rett syndrome are summarized in: http://mecp2.chw.edu.au/mecp2/index.php	Amir et al., 1999
MBD1	Prostate cancer	Upregulated	Patra et al., 2003
MBD2	Breast cancer	Upregulated	Billard et al., 2002
MBD3	Glioblastoma	Upregulated	Schlegel et al., 2002
MBD4	Colorectal cancer Endometrial cancer Pancreas cancer	Frameshift mutation Frameshift mutation Frameshift mutation	Riccio et al., 1999
KAISO	Colorectal cancer	Upregulated	Lopes et al., 2008
ZBTB4	Neuroblastoma	Downregulated	Weber et al., 2008
ICBP90	Non-small-cell lung cancer	Upregulated	Daskalos et al., 2011
NIRF	Lung cancer	Upregulated	He et al., 2009

Although the first patients were described in 1966 by Andreas Rett (Rett, 1966), more than 30 years passed before mutations within the *MECP2* gene located in Xq28 were identified as the cause of the neurological disorder (Amir et al., 1999). The most frequent mutations observed in patients suffering from RTT are missense mutations that cluster within the MBD (aa 78–162), as well as nonsense mutations primarily found within the TRD (aa 207–310; Christodoulou et al., 2003). In *Xenopus*, missense mutations R106W, R133C, F155S, and T158M were shown to reduce the binding ability of Mecp2 to methylated DNA (Ballestar et al., 2000). Studies in mouse cells showed that the majority of MBD-related missense mutations affected the heterochromatin binding and/or clustering ability of Mecp2 (Agarwal et al., 2011). By artificially targeting chromatin binding deficient Rett mutants (R111G, R133L, and F155S) to constitutive heterochromatic regions, however, Casas-Delucchi et al. (2012) revealed that some of these mutations exclusively affect the chromatin binding but not linking ability. Mutations within the TRD have been shown to influence protein–protein interactions. In knock-in mice bearing the common RTT mutation R306C, neuronal activity fails to induce T308 phosphorylation, a PTM required to suppress the interaction of Mecp2 with the co-repressor complex NCoR. Accordingly, R306C mutations result in persistent association of both proteins leading to decreased induction of a subset of activity-related genes (Ebert et al., 2013; Lyst et al., 2013). In addition to missense and nonsense mutations, reading frame shifts and C-term deletions were shown to give rise to RTT. Mice bearing a truncating mutation similar to those found in RTT patients showed normally localized Mecp2 proteins (Shahbazian et al., 2002). Histone H3, however, was hyperacetylated indicating abnormal chromatin architecture and misregulated gene expression (Shahbazian et al., 2002). Moreover, Muotri et al. (2010) identified increased susceptibility for L1 transposition and genome instability in RTT patients with truncating mutations.

In addition to RTT, Mecp2 was implicated in other neurological diseases, including Hirschsprung’s disease, autism spectrum disorder, schizophrenia, Prader-Willi, and Angelman syndromes (Carney et al., 2003; Shibayama et al., 2004; Nagarajan et al., 2006; Loat et al., 2008; Ramocki et al., 2009; Zhou et al., 2013).

More recently MBP have been associated with several types of human cancers (**Table 6**). While Mecp2 was overexpressed in estrogen receptor positive human breast cancer (Muller et al., 2003), MBD1 mRNA and protein levels were increased in prostate cancer (Patra et al., 2003). Accordingly, Patra et al. (2003) proposed MBD1 as the major cause of hypermethylated chromatin regions in prostate cancer through the recruitment of HDAC1/2 and subsequent histone deacetylation. MBD2 mRNA level were shown to be significantly elevated in benign tumors of the breast and correlated with tumor size of invasive ductal carcinomas, the most common type of breast cancer (Billard et al., 2002). Accordingly, upregulation of MBD2 was proposed to be associated with breast cell proliferation (Billard et al., 2002). Increased expression of MBD3 and MBD4 were associated with malignant glioma of the brain, and the grade of malignancy correlated with MBD3/4 expression level (Schlegel et al., 2002). Furthermore, frameshift mutations of MBD4 have been identified in colorectal, endometrial and pancreatic cancer with microsatellite instability (Riccio et al., 1999). MBD4 mutations consisted of 1- to 2-bp deletions or 1-bp insertions that caused frameshifts and premature stop codons. The resultant truncated MBD4 proteins were predicted to be non-functional, as they lack the C-terminal catalytic domain, whereby genomic instability was proposed to steadily increase (Riccio et al., 1999). As a regulator of target genes of the canonical and non-canonical Wnt pathway, Kaiso was shown to mediate silencing of tumor suppressor genes CDKN2A and HIC1 in Wnt-driven human colon cancer cell lines (Lopes et al., 2008). Kaiso depletion induced expression of tumor suppressor genes without altering DNA methylation levels (Lopes et al., 2008). As a result, colon cancer cells became susceptible to cell cycle arrest and cell death induced by chemotherapy (Lopes et al., 2008). Accordingly, Lopes et al. (2008) suggested Kaiso as a methylation-dependent oncogene that represses hypermethylated tumor suppressor genes. ZBTB4 expression was shown to be downregulated in advanced stages of human neuroblastoma and multiple human solid tumors (Weber et al., 2008). As a repressor of the *P21^CIP1^* gene, an inhibitor of the Cdk2 kinase, ZBTB4 usually blocks cell cycle arrest in response to p53 activation (Weber et al., 2008). Consequently, loss of ZBTB4 inhibits apoptosis and favors long-term survival of affected cells (Weber et al., 2008). In tumors, where many promoter-associated CGIs are hypermethylated, maintenance of methylation plays a major role. Accordingly, elevated levels of ICBP90 were shown to control cell cycle through maintenance of promoter methylation at CDK2A and RASSF1 in non-small-cell lung cancer (Daskalos et al., 2011). Finally, decreased expression of let-7a miRNA in lung cancer was shown to result in elevated NIRF and reduced P21^CIP1^ protein level, thereby most likely contributing to lung carcinogenesis (He et al., 2009).

## Concluding Remarks

**FIGURE 8:**
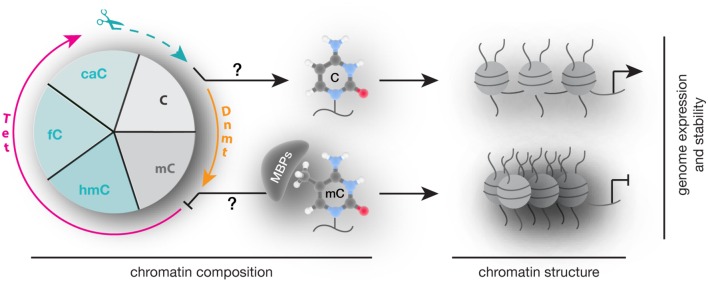
**Writing, reading, and translating DNA modifications.** Graphical summary of how DNA modification writers, readers, and translators can impact on chromatin composition, structure (nucleosomes are represented as balls, DNA as line) as well as genome expression (arrow represents active promoters) and stability.

In summary, alterations in 5mC writers, readers, and modifiers that affect their level, PTMs, ability to bind and/or modify DNA and protein interactions are each and all potential mechanisms contributing to altered chromatin composition and structure as well as genome activity and stability (**Figure 8**) and contribute to an overwhelming variety of human diseases. Despite intensive research, genotype–phenotype connections have been generally difficult to establish and subsequent studies are urgently needed to elucidate potential strategies for diagnostic and therapeutic applications.

## Author Contributions

All authors listed, have made substantial, direct and intellectual contribution to the work, and approved it for publication.

## Conflict of Interest Statement

The authors declare that the research was conducted in the absence of any commercial or financial relationships that could be construed as a potential conflict of interest.
